# Multisegmental tubercular spinal epidural abscess

**DOI:** 10.11604/pamj.2013.14.2.2268

**Published:** 2013-01-02

**Authors:** Hassan Baallal, Brahim El Mostarchid

**Affiliations:** 1 Department of Neurosurgery, Mohammed V Military Teaching Hospital, University of King Mohammed V Souissi, Rabat, Morocco

**Keywords:** spinal epidural abscess, multisegmental tubercular, tuberculosis, surgical decompression, antituberculosis chemotherapy

## Image in medicine

Tubercular spinal epidural abscess (SEA) is is a devastating infectious disease. Its presence without associated osseous involvement may be considered an extremely rare scenario. We present a rare case of tubercular SEA complicated by paraplegia in an immune-competent 58-year-old male patient. MRI shows a multisegmental posterior collection of epidural fluid extending from C7 to L2 vertebral level and displaces the ventrally located thecal sac, without any evidence of vertebral involvement. The patient made an uneventful recovery following surgical decompression and antitubercular chemotherapy. The diagnosis was confirmed by histopathological demonstration of Mycobacterium tuberculosis in drained pus. Such presentation of tubercular SEA has not been reported previously in the English language based medical literature to the best of our knowledge.

**Figure 1:**
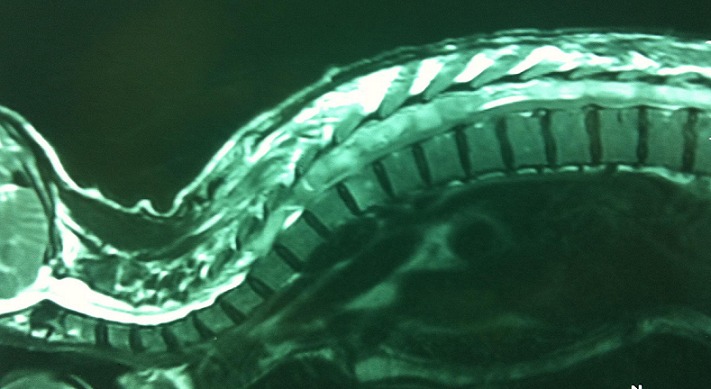
Sagital MRI in T2 shows a multisegmental posterior collection of epidural fluid extending from C7 to L2 vertebral level and displaces the ventrally located thecal sac

